# A Perspective of Non-Fiber-Optical Metamaterial and Piezoelectric Material Sensing in Automated Structural Health Monitoring

**DOI:** 10.3390/s19071490

**Published:** 2019-03-27

**Authors:** Venu Gopal Madhav Annamdas, Chee Kiong Soh

**Affiliations:** 1 School of Electrical and Electronic Engineering, Nanyang Technological University, 50 Nanyang Avenue, Singapore 639798, Singapore; 2 School of Civil and Environmental Engineering, Nanyang Technological University, 50 Nanyang Avenue, Singapore 639798, Singapore; csohck@ntu.edu.sg

**Keywords:** metamaterial, robot, piezoelectric, sensors, non-fiber optics, automation, structural health monitoring

## Abstract

Metamaterials are familiar in life sciences, but are only recently adopted in structural health monitoring (SHM). Even though they have existed for some time, they are only recently classified as smart materials suitable for civil, mechanical, and aerospace (CMA) engineering. There are still not many commercialized metamaterial designs suitable for CMA sensing applications. On the other hand, piezoelectric materials are one of the popular smart materials in use for about 25 years. Both these materials are non-fiber-optical in nature and are robust to withstand the rugged CMA engineering environment, if proper designs are adopted. However, no single smart material or SHM technique can ever address the complexities of CMA structures and a combination of such sensors along with popular fiber optical sensors should be encouraged. Furthermore, the global demand for miniaturization of SHM equipment, automation and portability is also on the rise as indicated by several global marketing strategists. Recently, Technavio analysts, a well-known market research company estimated the global SHM market to grow from the current US $ 1.48 billion to US $ 3.38 billion by 2023, at a compound annual growth rate (CAGR) of 17.93%. The market for metamaterial is expected to grow rapidly at a CAGR of more than 22% and the market for piezoelectric materials is expected to accelerate at a CAGR of over 13%. At the same time, the global automation and robotics market in the automotive industry is expected to post a CAGR of close to 8%. The fusion of such smart materials along with automation can increase the overall market enormously. Thus, this invited review paper presents a positive perspective of these non-fiber-optic sensors, especially those made of metamaterial designs. Additionally, our recent work related to near field setup, a portable meta setup, and their functionalities along with a novel piezoelectric catchment sensor are discussed.

## 1. Introduction

In the last half-a-century, there has been an exponential surge in the volume of research on SHM with at least 17,000 papers published in the last decade alone [1]. The major reason can be attributed to the enormous rise in the global demand for (a) wired and wireless sensing techniques, (b) miniaturization of analyzers, (c) energy harvesting of sensors and actuators, and (d) robotic applications. The efficiencies of many SHM techniques improved rapidly due to advancements in the designs of sensors, especially those made of smart materials [2,3,4,5]. Most such sensors can be classified as either FO sensors [6,7] or non-FO sensors [8,9] or their combinations [1,10,11]. The input of EM radiations or electrical power or magnetic field either remotely or directly to most smart material sensors via analysers/devices, produce diagnosable output signals. If such sensors are attached to or embedded in any CMA structures, they can probe the structures’ health in either continuous real time domain or discontinuous frequency domain [12]. Any deviation in the output signal from the healthy stage signal indicates possible anomalies in the CMA engineering structure under investigation. However, handling of (a) heavy and bulky input-output analysers or devices, (b) various types of smart material sensors from their installation to collection of usable output data, and (c) post processing output signals, are all important tasks requiring labour or engineers. The advent of automation, especially the application of robots coupled with miniaturized devices has greatly reduced such human tasks and increased the demand for qualitative SHM. Recently, Technavio analysts, a well-known market research company estimated the global SHM market to surge from the current US $ 1.48 billion to US $ 3.38 billion by 2023, at a CAGR of 17.93% [13]. 

This shows that the demand for SHM, especially the popular smart materials such as FO sensors and their operational designs, viz., (a) (FBG) sensors, and (b) modulated sensors are on the rise [14]. Usually the modulations are induced in intensity, wavelength, phase, polarizations, and gratings. Similarly, adaptable and innovative designs of non-FO smart materials such as metamaterial and piezoelectric materials are also on the rise. For example, the market for metamaterials and piezoelectric materials are expected to grow rapidly at a CAGR of more than 22% and 13%, respectively [15,16]. Furthermore, the global automation and robotics market in the automotive industry is expected to post a CAGR of close to 8% [17]. The fusion of such smart material sensors with automation can increase the overall market enormously and shape the future to a great extent [18]. Thus, this invited review paper presents a positive perspective of non-FO materials especially metamaterials with brief discussion on characteristic features of EM near field and far field; and evolution of EM metamaterial designs with relevance to these fields for SHM applications. Additionally, our recent work related to near field setup using a few near field metamaterial designs, a portable meta setup, and their functionalities along with a novel piezoelectric catchment sensor are discussed.

## 2. Non-FO Smart Materials: Metamaterials 

### 2.1. Positive Perceptive of Metamaterials in SHM

**Figure 1:**
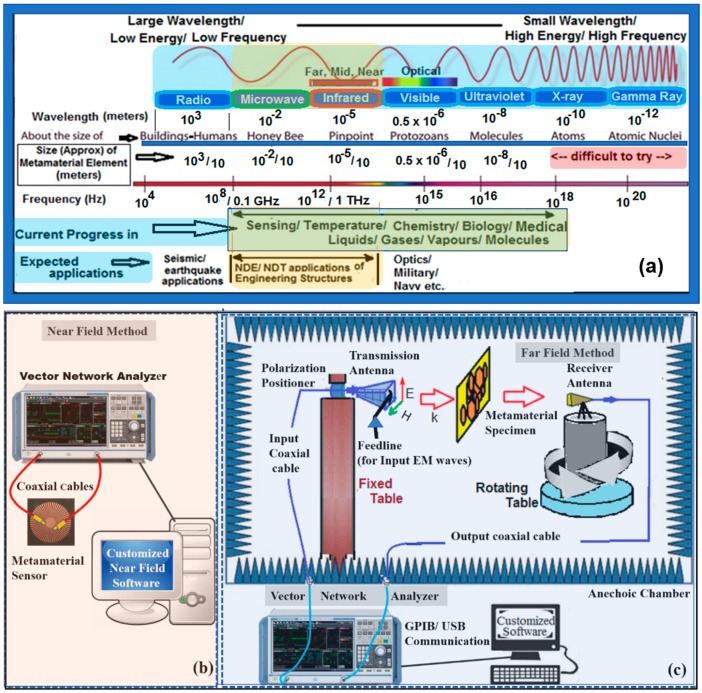
Electromagnetic field (**a**) spectrum, (**b**) input–output of metamaterial in far field, and (**c**) near field.

Recent introduction of designed or customized metamaterials have shown to improve the sensitivity of several biomedical and human health sensors [19,20], or if required, substituted the poor performing sensors [21,22]. The metamaterials are fabricated by artificially organized materials, which are engineered to have properties that are not available in nature. Thus, they are assemblies of several individual elements such as gold or copper or dielectric, and are arranged in a pattern as a single layer or stacked as multiple layers with intermediate separating layers on the surface of or embedded inside the base substrate [23]. These designs are developed to create desired material response based on near field or far field EM applications [24]. Their precise shape, geometry, size, orientation, and arrangement can affect the EM light or sound waves in a manner not observed in natural materials [25]. However, these EM waves are to be regulated, i.e., polarized in a single plane via polarizing filter or a panel with slits. These slits regulate the intensity of the polarized light beams. The polarization adopted so far in various applications are of four types, viz., horizontal, vertical, circular, and elliptical. However, only horizontal and vertical polarizations has been adopted in several applications of SHM [26]. EM waves generate energy that is produced by oscillating electric (E) and magnetic (H) disturbance in perpendicular planes, or by the movement of electrically charged particles known as electrons that emits as waves or radiations from a light source [27]. The energy can be grouped into categories based on the wavelength in the EM spectrum (Figure 1). The electric and magnetic waves that travel (wave propagation K) perpendicular to each other has certain characteristic features which include amplitude, wavelength, and frequency. These individual groupings form the basis for several metamaterial applications in near and far fields.

Figure 1a also shows the approximate size of element, the present progress made in the field, and possible future applications. Thus, the elements required for seismic application are many times larger than the elements required in the ultraviolet range. For example, on one end of the spectrum, we must use big stones or boulders as elements to divert or alter earthquake waves. In the middle of the spectrum, we need micro structure in the order of a few hundred of nanometers to divert visible waves. However, on the other end of the spectrum, we need much smaller size elements which cannot be developed at present. Thus, we notice several key facts as we move from left to right in the EM spectrum, such as wavelength and the size of the metamaterial element decreases from radio to gamma range. This implies that if the input EM wave length is large, then it finds application in CMA engineering for SHM and if it is too tiny then it finds applications in biology/chemistry. The cost of analyzers and faculties also increase as frequency wave of excitation increases from left to right in the spectrum. Nonetheless, as SHM applications need relatively larger element sizes than the sciences, we can limit the cost of our analyzer/devices to a greater extent. 

Figure 1b,c show the near field and far field experimental setups, usually adopted for execution of metamaterial designs. The near field and far field are the EM regions around an object such as a transmitting antenna which results in radiation scattering off in the vicinity. ‘Near field’ behavior, which is generally non-radiative dominates close to the antenna or scattering object, whereas ‘far field’ behavior dominates at larger distances. Generally, radiation decreases as distance increases in the near field region, whereas it decreases as the square of distance in the far field region. There is no clear distinction between the near and far fields, but in the literature the differences between these fields are decided based on (a) the wavelength of the scattering (transmitted) wave, (b) distance from the antenna, and (c) the largest dimension of the antenna [28,29]. 

The near field setup consists of a pair of tiny dipoles or antennas, where one is a source emitting antenna and the other is a transmission receiving antenna, placed nearer to the metamaterial sensor. However, source antenna can function as receiver if it is required to capture reflection signals as well. Thus, we can measure both the transmission and reflection signals in this setup. On the other hand, a far field setup consists of a pair of large antennas, which are used separately as a transmitter and a receiver, placed far from the metamaterial specimen but inside an enclosed anechoic chamber. This chamber consists of polarization positioner and a large source antenna for streamlining input EM radiations aimed to transmit along propagation direction, which eventually scatters around, and a part of it gets reflected and refracted. The percentage of reflection, transmission, and absorption depends on the properties of the metamaterial sample, the suspended medium or the engineering structure under investigation. However, only the transmission gets captured by the receiver antenna while the reflection does not go (or feed) back to the transmitter unlike the near field. The reflection plays an important role, especially for large surface structures. Thus, an engineer/scientist working in near field may collect more EM data as compared to the far field, a desirable feature for SHM. Additionally, set-up of a far field laboratory is very expensive as scattering radiation needs to be completely absorbed. Furthermore, the electric and magnetic fields can exist independent of each other in the near-field region [30,31], and we can measure electric fields using electric analysers and magnetic fields using magnetic probes or a common analyzer for both fields. One such electric and magnetic field measuring analyser is a VNA, a commercially available product for EM input supply and output measurements. This VNA can be used in near field and far field applications by changing or tuning it accordingly [32,33,34]. In either case, the output results in Z or S parameters that change/shift in the presence of any discrepancy/fault in the structure or medium. The impedance output is measured at lower frequencies in the order of Hz or K Hz [35], in which a total current or voltage measurement in sinusoidal frequency distribution is acquired at the input (reflected wave) port 1 or the output (transmitted wave) port 2 of a device or nodes of a VNA. However, at high frequencies (Giga Hz or Tera Hz) it is difficult to measure the total current or voltage because high energy is associated at higher wave frequencies. Energy increases as we move from left to right in the EM spectrum, which will damage the circuits of analyser if Z-parameters are measured. Hence S-parameters are generally measured instead of the impedance. 

The choice of adopting near field or far field depends on many factors such as EM radiation wavelengths, application, expertise, funding, etc. For example, kitchen oven can be a very simple model of the near field EM appliance where EM radiations in the microwave range heats the food due to the tiny dipole antennas closer to the food items, which may take longer time in the far fields with large antenna placed at a farther distance from the food. Thus, after studying various such pros and cons of EM fields, we have adopted near field experimental setup for our SHM applications in the microwave range as it avoided expensive anechoic chamber and can provide on-site monitoring.

### 2.2. Metamaterial Designs

In this world, there exist several naturally formed materials or organized materials or gases which create illusions by deflecting EM radiations due to their negative permeability and permittivity such as mirages [36]. The initial contributions to understanding such a behavour were made about five decades ago by V. G. Veselago who predicted that the materials with both negative permittivity and permeability are theoretically possible to be constructed in future [37]. However, the major contribution only came three decades later from John Pendry group, especially towards the development of various far-field based metamaterial designs. They proposed periodically arranged wired based TW structural design which depicts the negative value of effective permittivity [38]. It was shown that the structure had a lower plasma frequency than the wave in the microwave regime. Similar behavior was achieved using an array of SRR design [39]. In 2000, Smith et al. demonstrated a new LHM which produced simultaneously negative permittivity and permeability, and carried out microwave experiments [40]. Later, Shelby et al. showed negative refraction experimentally for the first time using a metamaterial design comprising of repeated SRR unit cells and copper strips [41,42,43]. In 2005, Wu et al. proposed three types of SRR designs, viz., symmetrical ring, omega, and S shaped sensors [44]. Furthermore, these designs have smart cavities or spacings between the elements, which are capable of altering EM waves by blocking, absorbing, enhancing, or bending. Such designs can be considered as part of a ‘family of smart materials’ useful for SHM.

**Figure 2:**
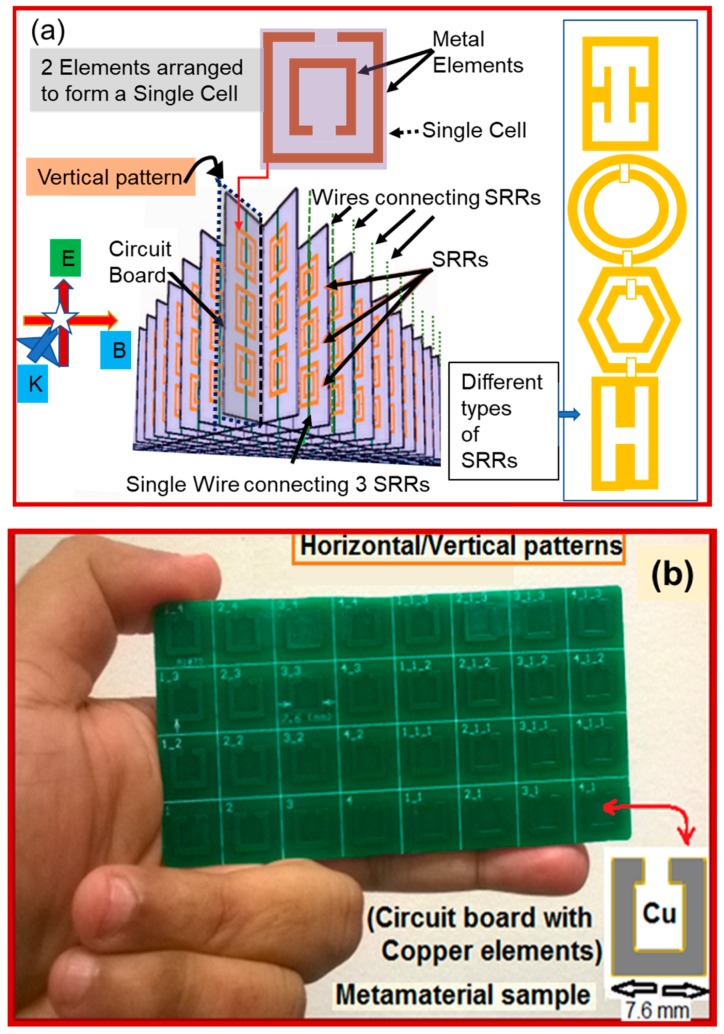
Typical examples (**a**) various orientations of SRR on a circuit board, and (**b**) rectangular metamaterial sample.

Figure 2a shows an example of a typical SRR design consisting of vertical patterns where each pattern has three-unit cells. Each cell consists of an inner metal square with a split on one side, embedded in an outer square with a split on the other side. The SRRs are on the front surfaces of the square grid as shown, whereas the single vertical wires are on the back surfaces [35,40,45]. All types of SRR single cells generally have a pair of enclosed loops with splits in them, at opposite ends, as shown in the figure. The loops are made of non-magnetic metal like copper with a small gap between them and they can be concentric, square, rectangular, or gapped as needed. Figure 2b shows an example of an embedded copper element of 7.6 mm × 7.6 mm on a circuit board sample of about 11 cm × 5 cm × 2 mm, known as inclusions. Various other designs are possible by merely changing the element shape and size [46]. 

Most of such designs and their hybrids [39,47,48] gained importance academically, especially in the UK (Pendry group at Imperial college), which expanded from USA (especially Smith group at Duke University) to Asia (especially in China, Singapore, and Japan) for applications in electronics and sciences; but negligible for SHM. They were applied in super-lenses [48,49], slow light [50,51,52], data storage [53], optics [54], cloaking/invisibility [55,56,57,58], sub-wavelength imaging [59], gradient negative index lenses [60], perfect absorbers [61], metasurface [62], photonic topological insulators [63], and so on. Until now, a variety of metamaterial designs have been successfully developed for a fairly wide range of the EM spectrum (Figure 1a) such as microwave, infrared and optical frequencies [64,65,66]. However, they were mostly adopted for non-CMA structures, and their latest entry for SHM of CMA structures [67,68,69,70,71,72] started attracting engineering groups like ours. This is because of their swift output variations to the geometrical changes in the unit element or assemblies of elements [73], making them suitable for monitoring of ‘strain’, a common engineering problem for most CMA structures. However, metamaterials do not have any standard designs unlike the existing smart materials or nanomaterials to cater the needs of CMA academia or industry; hence, there is a lot of scope for future research in design and applications. To encourage metamaterial designs in SHM applications, we need to address several important challenges, such as to find (a) suitable designs based on the dimensions of the CMA structure (small, medium, or large); and (b) method of application (far field, near field, and semi near/far fields). These factors decide future course of action of not just these metamaterial sensors, but for any new material or sensor which can be built or discovered. Our literature review based on available previous designs and applications [74,75,76,77] suggests that the metamaterial elements required in the construction of a pattern can be between 1/8 to 1/12 of the maximum input wavelength of interest. Furthermore, the efficiency of existing sensing qualities can also be improved using deposition of metamaterial elements in a pattern on some type of traditional sensors; research on this is still ongoing [73,74,75,76,77,78]. Unfortunately, no single design or deposition of a design or SHM technique could solve several complicated engineering issues by itself. A combination of such designs and their integration with other SHM techniques are thus necessary.

#### 2.2.1. Some Examples of Metamaterial Designs in SHM and Future Path

Different criteria are used to differentiate between different applications, for example, application in civil engineering is different from application in biology. The first criterion is the input/output wavelength (or frequency) and the other is the design (pattern and element sizes) of metamaterial. In the last 10–12 years, researchers and designers from material, electrical, and electronic engineering are trying to establish their designs in the CMA engineering market apart from the regular market in chemical, biological, and medical sciences. CMA engineers and scientists need to understand the pros and cons of these designs or sensors before recommending them. This section presents some such designs available in the literature for strain applications, and the path forward.

A) Designs by Melik et al. [79]

Measuring and reporting strain in structural components using telemetric methods represents a significant engineering challenge. About two decades ago, initial efforts in developing of metamaterial designs for SHM of CMA came from Melik group. They proposed metamaterial-based strain sensors, which are highly sensitive to deformation by improving their previous design architectures that were implemented for bio applications [4,80]. 

They developed a 5 × 5 SRR architecture which comprises of patterns along vertical and horizon directions similar to that shown in Figure 2b, but greatly reduced in dimensions. This metamaterial sensor comprises of a base silicon substrate of 0.1 μm thick Si3N4, which was deposited by plasma-enhanced chemical vapor deposition method. On the top of this substrate, a pattern of 0.1 μm thick ‘Au’ film was built using standard lithography, metal evaporation, and lift-off technique; to achieve an SRR structure with a 2220 μm outer length and a 1500 μm inner length. The unit cell length comprises of 2780 μm, and hence a 5 × 5 array of the SRR resulted in a total of 1.5 cm^2^ sized metamaterial sensor. Their experiment comprised of far field setup in an enclosed anechoic chamber (Figure 1c). The SRR array sensor was surface bonded using epoxy on a polyamide stick specimen for strain monitoring. This specimen was then subjected to a compressive load from 0 to 300 kgf. Technically, the compression resulted in a decrease of the dielectric area of the base substrate and capacitance; whereas an increase in spacing between the Au metal elements or between the inner and outer SRRs in a unit cell resulted in an overall increase in the resonance frequency. As stated earlier, in a far field setup only transmission signals are possible, which are acquired in a narrow range of 12 to 12.6 GHz. They reported a sensitivity of 109 kHz/kgf or 5.148 kHz/microstrain with error of 200 microstrain and a frequency shift of 109 kHz for every 1 kgf compressive force, or 5.148 kHz shift for every microstrain increment. 

Later, the same research group proposed a flexible metamaterial design with increased sensitivity, suitable for attaching on any curved or nonplanar surface. In their previous design they used a silicon substrate, whereas in this design they used flexible substrate (Kapton) tape as base material. This is a vacuum tape of the polyimide type with a great heat resistance, which is generally used in fabrication and packaging. This tape contains silicone adhesive on the back side that does not leave any residue when the tape is removed [71,81]. This new design has greater sensitivity and a more linear response to compressive loading compared to their previous designs. This exhibited a 6-fold increase in sensitivity and a 16-fold reduction in error. Frequency shifts by 0.292 MHz for every 1 kgf compressive force.

Later, the same research group presented another design for compressive load monitoring. Three sensors made of this design were surface bonded one each on three different structures made of a hard material (cast polyamide), semi-hard material (derlin), and a soft material (polyamide). Compressive loading tests from 28 to 271 kgf were carried out for all these specimens [82]. The hard material (high Young’s modulus) shown a lower gradient in the frequency shift, whereas the soft material (low Young’s modulus) shown larger gradient. The sensitivity of the metamaterial sensor was reported as 0.0543, 0.0577, and 0.119 MHz respectively for every 1kgf compressive force. A one microstrain change resulted in 2.348 × $10^{-3}$ MHz, 2.224 × $10^{-3}$ MHz, and 3.224 × $10^{-3}$ MHz, respectively.

Path forward: The work carried out by the Melik group [79,80,81,82,83] are preliminary in nature and needs further investigations. They tested their designs on simple specimens in far field setup; which should be extended for real CMA engineering specimens. These designs should be adopted to monitor metals like steel with significantly high Young’s modulus and non-metals like concrete or timber with lower Young’s modulus. 

Furthermore, without massive commercialization, development of such sensors for individual research groups is costly, as it requires facilities such as chemical vapour deposition, metal evaporation, lithography, and lift-off techniques, apart from building expensive anechoic chamber. The frequency range adopted falls in the Giga Hz, on the right side within the microwave spectrum (Figure 1a). This can be useful for NDT adaptable in isolation, but people using CMA structures may wish to use the structures even during the period of monitoring. However, researchers can modify the size and shape of SRRs which forms the characteristic basis for highly sensitive designs. As stated earlier, lack of commercialization can enforce CMA engineers/scientists to opt for alternative designs, which they may have to fabricate based on complexity of the engineering structure under inspection, and they could be adopted in open air based near fields so that people can still use the engineering structure even while monitoring it.

B) Design by Li et al. [76,84,85]

Two metamaterial designs were built by Li et al, which were subjected to frequency excitation of lower order Giga Hertz (>10 GHz) that resulted in output signals suitable for strain monitoring. They preferred to call these designs as “metamaterial inspired” strain sensors rather than metamaterial strain sensors as their designs do not resemble the well-established SRR designs. Nonetheless, the concept adopted is the same as discussed in the previous designs. Instead of SRRs these researchers used simple I-shaped unit cells, suitable for single-axis strain sensing, and the other is a crossed-I (or +) shaped unit cell for dual-axis sensing. The elements of unit cells are made of Au, and the substrate adopted is PDMS. Both these designs comprise of several such unit cells that are directly surface bonded to a PDMS layer to make a metamaterial sensor which can then be attached to any test specimen. Compared to the silicon substrate (base material) as mentioned previously by Melik et al. [4,79,80,81,82,83], the PDMS substrate is not only cost effective, but it is a self-adhesive tape with high flexibility and excellent elastic properties. Furthermore, the sensitivities of these sensors are high, owing to the high surface tension of PDMS substrate. Both of these sensors have a maximum sensitivity of 107 GHz per microstrain under the applied stress. These sensors can even be attached to smooth surfaces without any additional adhesive, which facilitates direct strain transfer from engineering structure under inspection to the sensor to measure more direct strain. 

Path Forward: The designs of Li group are smaller compared to the earlier SRR designs. Furthermore, they can be varied by changing the dimensions of I-shaped and crossed I-shaped unit cells and the spacing between them. As stated earlier, the size of the metamaterial element reduces as we move from left to right in the EM spectrum, which implies extreme heat generations that affect the measuring devices. Thus, the designers should make their designs resistive to higher temperatures if they aim to build metamaterials for higher frequencies with smaller element sizes and subsequent smaller sample sizes. However, this enforces their application only in the far fields.

The designs of Li group, or any small sized elements, can be adopted for monitoring of mechanical and aerospace engineering components or smaller civil engineering components in the far field setup. However, rugged civil infrastructures and most large mechanical or aerospace structures need open air acquisition as they cannot fit inside anechoic chambers. Thus, it is desirable to adopt a relatively larger element sizes for outdoor applications via near field setup using microwave range either in the Giga Hz or Tera Hz range. 

C) Design by Ozbey et al. [34,86,87]

The Ozbey group developed a design which can be applied in a partial near field setup unlike the previous two cases. This is a comb-like nested SRR (or simply named as N-SRR) probe with metal strips, first proposed by the same group in 2010 [83]. This is not exactly a strain measurement design, but developed uniquely for tracking separation, another common engineering problem near the joints of engineering structures. This comb-like sensor consists of two identical parts separated by a distance and connected to each other via a thin flexible wire. These two parts are free to move with respect to each other. This N-SRR was fabricated over a footprint of 47 × 47 mm^2^ on a single-sided Rogers Duroid substrate with a relative dielectric constant of 3.2 and a thickness of 0.508 mm. A near field effect can be developed when this N-SRR probe is placed closer to a large antenna. The technical aspects of design can be obtained from their articles [34,86,87]. 

In their experiments, the input EM wave as well as the output were handled by the same antenna. This implies that the measurement can be obtained at the source/incident port 1 and hence $S_{11}$ is obtained which was otherwise not possible in far field condition. However, their antenna is much wider with a feed line (to supply input) similar to the feedline of box/horn antenna commonly used in far field applications.

Their experiment was conducted in steps, where a N-SRR probe was first attached to a standard steel rebar specimen. Later, a compressive force is applied linearly from 0 to 900 kgf where the elastic region and plastic region were monitored [86]. In the next stage, a 4-cm thick concrete block was placed between the antenna and the receiver probe. The receiver was found to function effectively even through a concrete block [34]. This design exhibited a relatively high resolution (<1 μm) and sensitivity (>12.7 MHz/mm) over a wide distance of separation. 

Lately, this design was adopted to monitor a reinforced concrete beam in a simply supported condition. The N-SRR sensors were attached on the reinforcing bars inside the beam, and the antenna was placed outside the beam [87]. The results of the experiment show that the plastic deformation region strain/displacement can be detected wirelessly. The detailed study, including the sensitivity, resolution, and stress–strain results can be obtained from their work [34,86,87].

Path forward: The main problem with this N-SRR sensor is its dimension, which is much larger in the planar direction than the rebar specimen. Even then, it was adopted to monitor compressive load [34]. Such a situation should be avoided as the sensor is expected to be smaller than the engineering structure under investigation. The next problem is with the thin flexible wire, connecting two identical parts, which can break during the monitoring. However, a flaw/discrepancy in a deeper location up to 4 cm inside a structure can be detected. Making this design suitable candidate for SHM of reinforced concrete structures.

Until now, we saw metamaterial designs which are surface bonded or embedded inside the host structure under investigation, for strain and separation monitoring. All these designs covered applications in the EM microwave range plus a short range to its left (M Hz range) and a short range to its right (Tera Hz range) [4,34,85,88,89]. However, several permutations and combinations of designs are possible, depending on the element type (metallic or dielectric), size of element, pattern, type of substrate, excitation frequency, and method of application. Hence, there exists a huge research scope for designers to build greater sensitive patterns or better elements. The designs can be taken care by the designers or commercial companies so that the CMA engineers can concentrate on adopting suitable designs for their applications. Thus, we carefully examined the pros and cons of a few designs, especially the near field designs which are our major field of research.

#### 2.2.2. Our Near Field Metamaterial Designs 

Apart from the above discussed designs of SRR, I, crossed I, and N-SRR, there exist several other designs built using SPPs and localized surface plasmons (LSPs) materials which were validated for several applications including a few specially in SHM [67,70,82,90,91,92]. Even these plasmon designs comprise of elements made of metal or plastic inscriptions on materials such as Rogers RT using PCB technique [68,93,94,95,96]. When these designs are subjected to EM waves in the microwave range, the output in the form of either progressive or surface waves is observed in the far or near fields. These designs can be used for various studies, including health diagnosis of biological and engineering structures [12,73].

However, it is not always possible to attach or embed such metamaterial sensors to every engineering structure under inspection. Furthermore, huge and continuous structures such as bridges, airplanes, machines, MRT stations, and trains, can be neither monitored in isolation chambers nor by big antennas in public places.

Hence, near field experimental setup, which does not require big antennas and anechoic chambers need to be developed. This method of application (see Figure 1b) can monitor any on-site engineering structure such as a bridge, MRT station, or train. Apart from the strain and separation measurements, we can extend metamaterials to various studies such as crack, damage, corrosion, and static and dynamic load monitoring. They can co-exist with FO based technologies that are mostly adopted for large engineering infrastructures [10,14,97]. 

Applications of metamaterial sensors or metamaterial inspired sensors in sciences, especially in biology and medical are well established, but they are still in the preliminary stages in CMA engineering, even after a decade of research efforts. Furthermore, each metamaterial sensor or design is discrete and as a multiple-metamaterial (network) sensing technique is not yet explored. 

‘Reshape and rethink’ is a phrase attributed to metamaterial designs by Lin et al. group [98]. Thus, considering all the aspects studied earlier, we developed a few simple near field based LSP designs inspired by the simplistic design of Shen group [91]. We considered factors such as their resemblance, shape, size, properties, sensitivity, and their performance in comparison to other leading smart material sensors, especially the piezoelectric type of sensors which are already in use for SHM [12,99].

We developed a radial-LSP design and a ‘microwave near field’ software [100,101]. Recently, a new longitudinal-LSP design is also introduced successfully by supplying near field EM microwaves to a force sensing resistor which generated diagnosable output surface waves. These types of LSP designs are highly non-fragile as they are manufactured as bi-material composites with metal inscriptions on flexible and ultra-thin dielectric or piezoelectric materials such as Rogers (RT5880), using PCB technique. They are bendable and twistable ranging from linear ($0^{0}$) to double bend ($180^{0}$) shapes [77,102].

**Figure 3:**
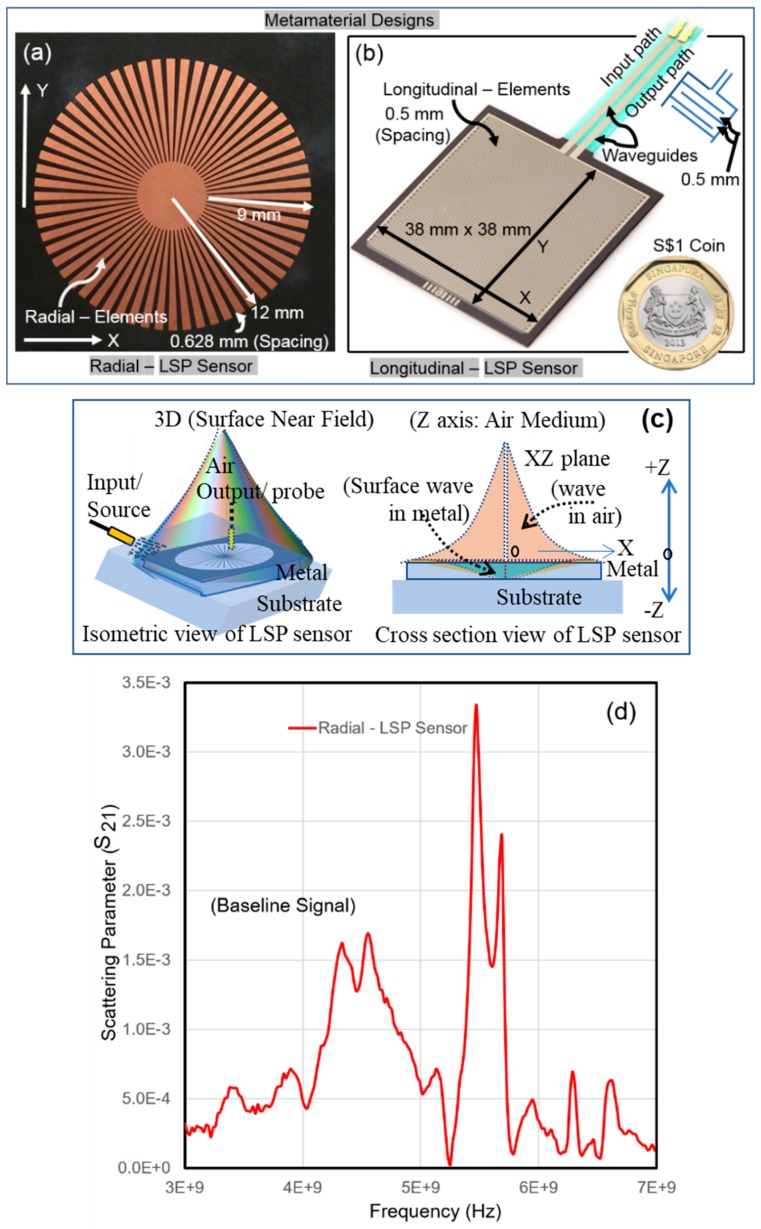

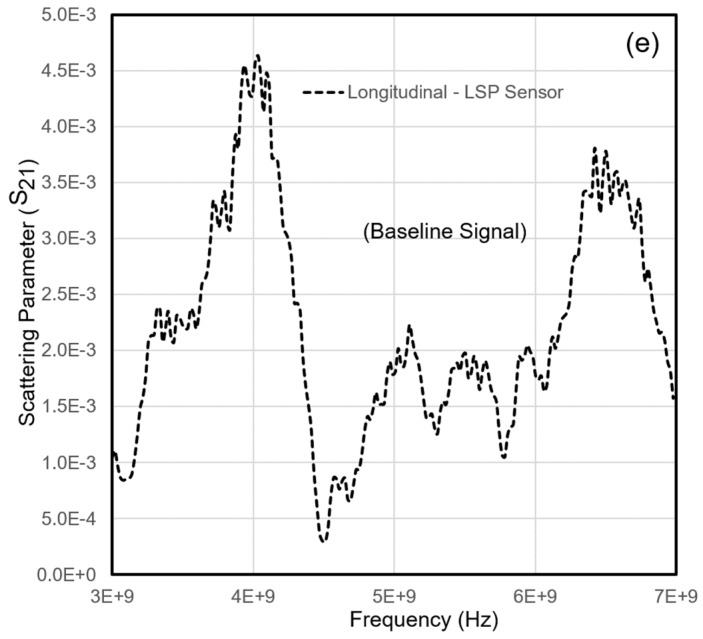
Representative LSP sensors and output (**a**) radial-LSP sensor, (**b**) longitudinal-LSP sensor, (**c**) probing in Near Field, (**d**) baseline signal of Radial-LSP sensor, and (**e**) baseline signal of longitudinal-LSP sensor.

Figure 3a shows a photo of our radial-LSP sensor, which comprises of periodic grooves with period of 1.256 mm and 0.628 mm air gap between neighboring strips (radial elements), etched on an 18 μm thick copper disk. This is engraved in a circular fashion on a 254 μm thick Rogers RT5880 substrate [77]. It is symmetrical in the X–Y plane and offers EM leakage-free application in the near field. Figure 3b shows a photo of our longitudinal-LSP sensor, which comprises of longitudinal metal strides and provides a 38 × 38 mm sensing area. 

The working principle of our designs involves application of a pair of dipole antennas where one is a point source dipole for input excitations and the other is a receiver dipole for output probing/receiving (see Figure 1b). Furthermore, Z-axis is normal to the X–Y/sensor plane, which starts from the interface of metal and air. The half space Z (< 0) is the zone in the metal while the other half space Z (> 0) is the zone in the dielectric material (air medium). The input EM wave on the sensor results in localized surface wave as output, that decays within the metal and the air medium above it, with two different non-linear curves along the negative and positive Z-axis as shown in Figure 3c. 

Thus, contactless acquisitions can be easily obtained above the sensor, i.e., Z > 0, where transmitted scattered parameters can be captured in the air medium by the receiver probe. However, in contact probing on the sensor, i.e., Z = 0, the input supply and output acquisition are both considered on the X–Y plane. In either case, a nonlinear output 3D zone is created in the space which evanescent (a) along Z-axis (Z > 0) in the air, and (b) from middle of the sensor to the edges of sensor in X–Y plane or in any plane parallel to X–Y plane. Compared to other LSP design, our LSP design provides two narrow input and output waveguides/inlets that facilitate smooth transfer of EM waves or electrical power supply or magnetic field.

#### 2.2.3. Brief Background of EM Surface Waves 

In general, the plane waves are eigen modes of propagating EM waves in bulk, linear and homogeneous materials. The fields $\vec{E}$ and $\vec{H}$ are phasor vectors of a plane wave in an isotropic dielectric material (such as air), which can be written as Ref. [103].

(1) $$\vec{E}=\bar{E}e^{i\vec{k}.\vec{r}}\;\mathrm{and}\;\vec{H}=\bar{H}e^{i\vec{k}.\vec{r}}$$

where $\vec{k}$ (=2π/λ) and $\vec{r}$ = $\sqrt{\left(x^{2}+y^{2}+z^{2}\right)}$ is the distance vector from the input source in space. Furthermore, the cross product

(2) $$\vec{k}\times \bar{E}=\omega \mu_{o}\bar{E}\;\mathrm{and}\;\vec{k}\times \bar{H}=-\omega \varepsilon_{o}\varepsilon \bar{E}$$

wave vector can be related to propagating wave and vertical axis (*Z*-axis) as

(3) $$\vec{k}=\kappa {\vec{u}}_{p}-ip_{m}{\vec{u}}_{Z}\;\mathrm{in}\;\mathrm{metal}\;(Z\;<\;0)\;\mathrm{and}\;\vec{k}=\kappa {\vec{u}}_{p}+ip_{d}{\vec{u}}_{Z}\;\mathrm{in}\;\mathrm{the}\;\mathrm{dielectric}\;(Z\;>\;0)$$

where ${\vec{u}}_{p}={\vec{u}}_{X}\cos\psi +{\vec{u}}_{Y}\sin\psi$ is a unit vector, ψ (0 to 360 degrees) is the angle with respect to the *X*-axis, and Re(κ)Im(κ) ≥ 0. 

In particular, these SPPs are EM surface waves in the visible/optical range that travel along a metal–dielectric (or air interface). This SPP involves both charge motion in the metal (known as surface plasmon) and EM waves in the air or dielectric material (known as polariton). A SPP will propagate along the interface until its energy is lost either due to absorption in the metal or scattering into the air medium [104,105]. However, to differentiate between the output signals of the far field in optical range and the near field in the microwave range, we adopted the nomenclature of ‘spoof SPP’ wave instead of SPP wave as suggested in the literature [106,107]. Generally, these SPP waves and spoof SPP waves are all surface waves with shorter wavelengths than the incident EM waves. However, their confinement in a region (known as sensing zone) and changes to the intensity in the confined zone at a specific location during the course of monitoring, forms the basis for all NDT applications either in sciences or engineering [108,109,110]. These waves comprise of electric field and magnetic field, perpendicular to each other. 

The electric fields for ‘transverse magnetic wave’ can be written in the simplest form of propagating spoof SPP wave, as

(4) $$\vec{E}={\bar{E}}_{m}e^{p_{m}Z}e^{i\kappa (x\cos\psi +y\sin\psi )},\;Z\;<\;0\;\mathrm{and}\;\vec{E}={\bar{E}}_{d}e^{-p_{d}Z}e^{i\kappa (x\cos\psi +y\sin\psi )},\;Z\;>\;0$$

where the subscripts m and d refer to the copper and air mediums, respectively. Similar expressions can be written for the magnetic field phasor H. 

If $P_{m}$ and $P_{d}$ have positive real parts, then both electric fields will decay as Z→±∞, indicating localization (within the vicinity) to the interference at Z = 0 (decays away from Z = 0). The confinement of such energized surface waves in the *X*–*Y* plane and the transportation of such energies until they fade out are important in the design of any surface plasmon materials [63,93,111]. 

#### 2.2.4. Experimental Measurement of EM Surface Waves 

The differences between the output scattered parameters obtained at healthy state and in several later conditions of the engineering structure form the application principle in SHM. We have earlier applied radial-LSP sensor in contact and contactless applications for monitoring the separation of two concrete specimens, pressure inside a cycle tube, the curvature of a metallic shell, and load and deflection monitoring of a beam using this principle [12,68,77,112]. The longitudinal-LSP design is our newest entry which has a larger sensing area than the radial-LSP design. Figure 3d–e show graphs of baseline output scattered parameter $s_{21}$ obtained using contactless probing of LSP sensors aimed to monitor an engineering specimen. The operation was carried out in the microwave range from 3 GHz to 7 GHz. The input and output dipole antennas were placed at 0.7 mm distance away from the sensors along Z-axis (Z > 0) at a central location. A closer look at the graphs show that the output magnitude of the longitudinal-LSP sensor is larger than the radial-LSP sensor. Furthermore, not only the change in sensor dimensions, but a different host engineering structure produces a different baseline signal [77]. Thus, indicating an existence of a unique health signal for every engineering structure using a considered sensor. Additionally, such LSP sensors are ultra-sensitive for monitoring ‘deformations’ of engineering structures, even if they are negligible in the order of 1/100th of a mm [12,77].

## 3. Our LSP Sensor 

**Figure 4:**
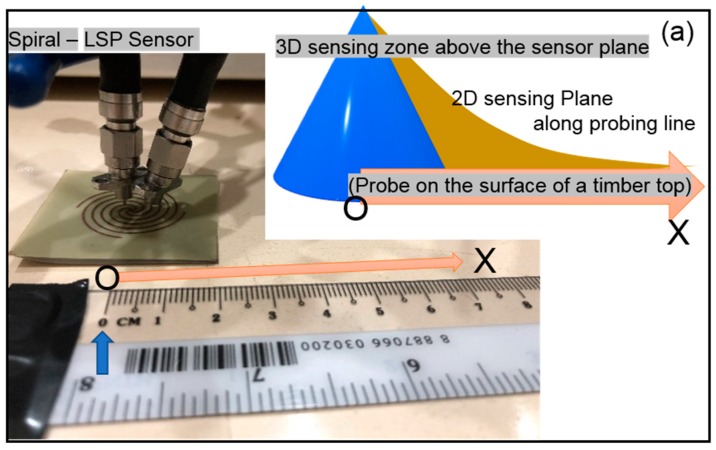

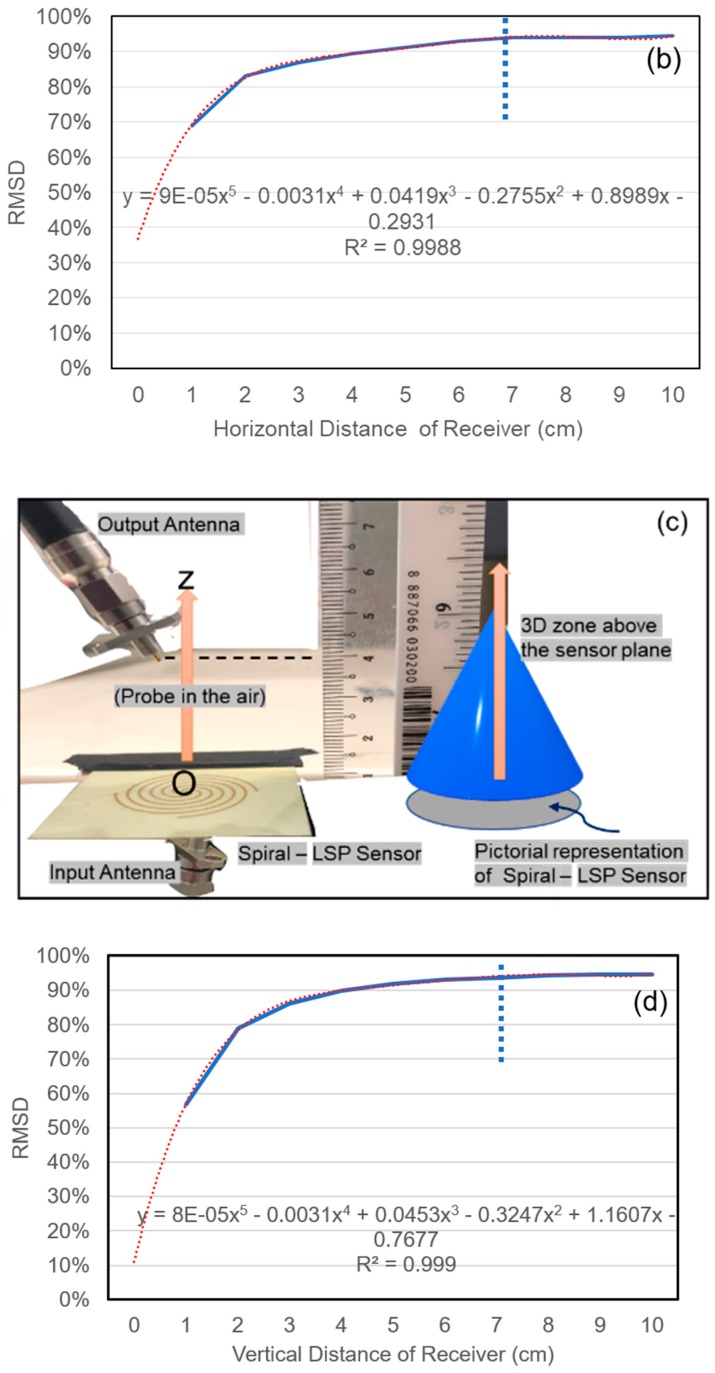
Representative LSP sensing zone study (**a**) horizontal radius of sensing, (**b**) RMSD variation of output signals in X-axis, (**c**) vertical radius of sensing, and (**d**) RMSD variation of output signals in Z-axis.

**Figure 5:**
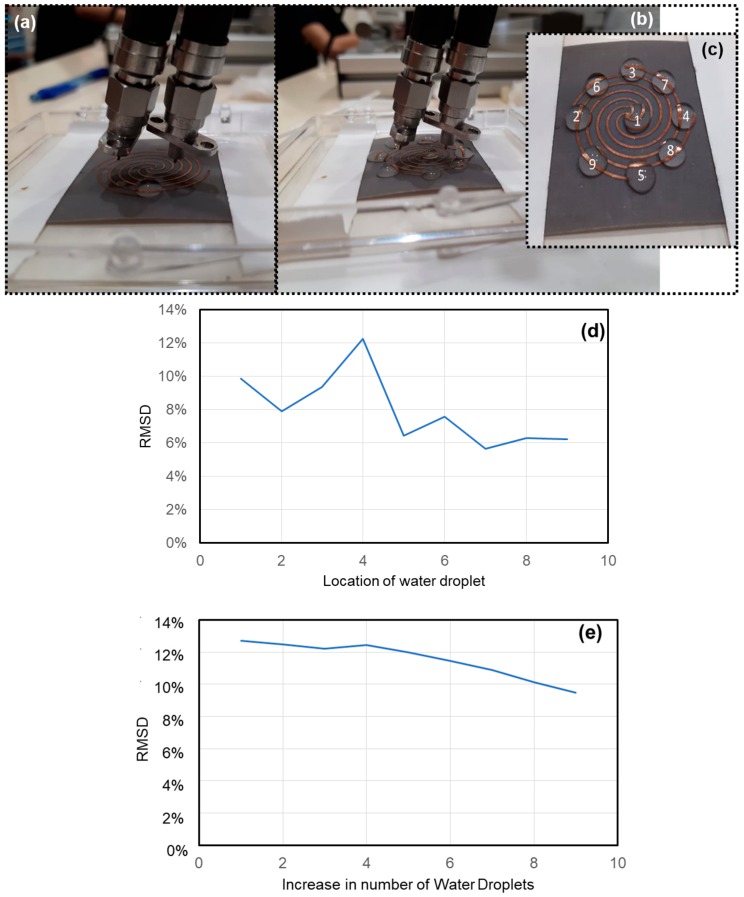
Spiral-LSP sensor and water effect (**a**) single water droplet on the sensor; (**b**) nine water droplets, dropped one after another; (**c**) order of dropping water droplets on the sensor; (**d**) RMSD variations when water droplets increase on the sensor; and (**e**) RMSD variations for water droplets at random locations.

Many types of LSP designs are possible for near field by merely changing the alignment of elements as similar to the SRR designs for far field. Thus, apart from radial and longitudinal patterns, there can be many more patterns such as spiral alignment as shown in Figure 4a. This type of spiral design was first manufactured by Huidobro along with Pendry and his group, but as a magnetic LSP design for meta-surface applications [113]. We adopted this spiral design to better understand a couple of crucial factors that are important for large scale application in SHM; especially, robustness and durability depend on the sensing zone and sustainability of the sensor to humidity and rain. Thus, we conducted several experiments on our spiral LSP sensor, as it has a lower metallic area, and easy to waterproof the top of the metal compared to other designs (Figure 4 and Figure 5). This section presents our laboratory experiments performed in near-field setup to evaluate this new spiral-LSP sensor using a popular RMSD based sensitive index. This RMSD sensitivity index provides quantitative differences between the output signals obtained at a baseline condition and those obtained at later stages. The quantity is a major yardstick for measuring sensitivity in CMA engineering [10,12,112,113,114,115]. This RMSD is given as

(5) $$\mathrm{RMSD}\;(\%)=\sqrt{{\sum_{i=1}^{N}\left({\left[y_{i}^{}\right]}_{j}-x_{i}\right)}^{2}/\sum_{i=1}^{N}{x_{i}}^{2}}\;\times \;100$$

where i = 1, 2, 3…, N represents sampling data points in the considered microwave band. $x_{i}$ is the baseline signal for all the N considered sampling points and $y_{i}$ represents subsequent signal obtained at the later stages for all the N considered sampling points. j represents the total number of later stages where subsequent output signals are obtained. The signal can be plotted as normal or logarithmic, but RMSD is always stated as a % variation of a later stage signal with the baseline signal. Thus, there can be many RMD values based on the number of signals (i.e., j) obtained for various conditions.

Such sensitivity indices are the most important assessment factor for any sensor because a mere output signal, i.e., scattered parameters such as shown earlier in Figure 3d–e cannot provide quantification. Furthermore, the raw output signals could be of interest only in physics or medical applications, but not for SHM [77]. Thus, any noise in raw output signals does not play an important role as long as it is uniform throughout the monitoring period. 

### 3.1. Identification of Horizontal and Vertical Sensing Radius of a Sensing Zone

The spiral design adopted for this study was manufactured by printing a 0.0035 mm thick copper metal on a 0.8 mm thick dielectric substrate with a possible spherical or elliptical sensing zone around it. The incident EM waves and the spill over radiations from the spiral-LSP sensor created spoof SPP waves along the scanning axis. To evaluate the probable sensing zone, the sensor was first placed on a low conductive timber platform.

#### 3.1.1. Horizontal Radius

The near field experimental setup (see Figure 1b) was adopted where input and output dipole antennas were placed at a height of 7 mm (Z > 0) above the sensor in the Z axis as shown in Figure 4a. Both antennas were connected to a VNA which was controlled by a near field software via coaxial cables. The figure also shows a schematically expected 3D sensing zone above the LSP sensor. The experiment began by instructing a 2D robot scanner with an automated movable arm, to carry and move forward the output ‘antenna/receiver’ horizontally above the sensor level (Z = 7 mm) along the positive *X*-axis in steps from 0 to 100 mm [77]. A baseline signal $s_{21}$ was first obtained from the output receiver antenna, and later the receiver was moved in steps to capture several outputs along the *X*-axis in the *X*–*Z* plane. In general, these output signals followed an exponential or a polynomial variation before it faded away along the *X*-axis. The quantification of variations in signals was calculated using RMSD equation as shown in Figure 4b. 

The curve becomes flat and parallel to the scanning axis after 7 cm of horizontal distance and beyond. The graph resembles a fifth order polynomial curve with the regression, R close to unity. Thus, the horizontal sensing radius can be considered as 7 cm but if the experimental specimen is a non-conductive concrete or conductive metal, then this horizontal radius will change as the travel path distance of spoof SPP waves depend on the conduction value of the host structure [116].

#### 3.1.2. Vertical Radius

The same experiment was repeated where the input antenna was placed 7 mm below the sensor, whereas the receiver was placed above the sensor and moved in the vertical plane, i.e., the scanning axis was along positive *Z*-axis. First a baseline signal was acquired and then the receiver was moved from 0 to 100 mm in steps of 10 mm as shown in Figure 4c. Figure 4d shows the RMSD curve, which becomes flat and parallel to the scanning axis after a vertical distance of 7 cm similar to the previous experiment. Once again, the graph resembles a fifth order polynomial curve with the regression, R close to unity. Thus, the vertical sensing radius can be considered as 7 cm in the air medium. 

Thus, both the horizontal and the vertical radius are equal, which indicates the presence of a spherical sensing zone around a flat sensor. However, the conductivity of timber, and the air medium are different resulting in different coefficients of *X* in the mathematical expressions as indicated in Figure 4b,d. Thus, the results could vary based on the type of experiment, method of application, and host material properties. 

### 3.2. Water Effect on the Sensor

In the recent past, Yang et al. group demonstrated that aligning water droplets in a pattern on a substrate resulted in a metamaterial absorber [117]. It is not uncommon to study water pattern, but we are interested in studying the influence of water droplets on the sensor rather than creating a new metamaterial design. Thus, we studied the water effect by dropping (a) a single 4 mL water droplet on the sensor in a random location, and (b) several water droplets in a pattern labelled from 1 to 9, as shown in Figure 5. The spiral LSP design was modified by replacing base substrate with a heavier Rogers RT5880 material of 254 μm thick, similar to the substrate of the radial LSP sensor.

Figure 5a shows the new spiral sensor placed in a small glass container, with a single water droplet on it. Figure 5b,c show the final photos of sensor with nine water droplets, which were dropped one after another on it. A baseline signal was initially obtained before the start of the experiments. 

In the first experiment, a water droplet was randomly dropped on the sensor and an output signal was obtained. Later the water droplet was wiped off using soft cotton before dropping another water droplet at another location. The process continued for eight more times to obtain eight more output signals. Figure 5d shows the RMSD curve representing the single droplet effect on the sensor at different random locations. It was observed that no two locations resulted in the same output signal. In the next experiment, water droplets were introduced one after another on the sensor in the order as labeled in Figure 5c. Each time, an output signal was obtained at increased water content on the sensor. Figure 5e shows a continuous RMSD variation due to increase in water content. The output signals obtained during increase in water content are different from the output signals obtained at random locations. Thus, it can be concluded from both experiments that the sensor is smart and works in the presence of rain or humid atmosphere unlike the other smart materials, especially the piezoelectric type sensors such as MFCs, piezoelectric diaphragms, PZT get short circuited and damaged in wet environments. 

### 3.3. Additional Role of Adjacent Sensor as Catchment of EM Waves

No single SHM technique can provide complete solutions to all complex health/aging problems faced by CMA structures [10,11,118,119]. Thus, CMA researchers opt for applications of several combinations of various SHM techniques to monitor strain, deformation, crack, fatigue, etc. for structures such as airplanes, machinery, pipelines, bridges, and buildings [120,121,122,123,124]. As stated earlier, some of the predominant sensors are glass-FBGs. There are many other smart sensors such as piezoelectric MFC, PZT, and diaphragm sensors [12,118]. Thus, it is quite common to encounter a piezoelectric sensor near another sensor of another SHM technique. Hence, an idea of having an additional role for an adjacent piezoelectric diaphragm within the vicinity of a radial LSP sensor led to the following experiment.

#### 3.3.1. Specimen

**Figure 6:**
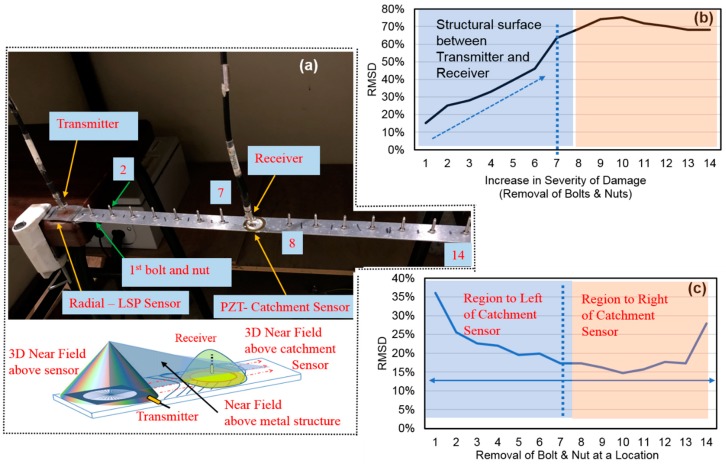
Introduction of catchment sensor and effectiveness in damage detection (**a**) photo and principle of dual sensor application, (**b**) increase in severity of damages on engineering surface between LSP sensor and catchment sensors and beyond catchment sensor, and (**c**) single damage on the specimen at various locations.

Figure 6 depicts two different types of defect monitoring experiments on a simply supported 1D aluminium strip of dimensions 60 × 2.5 × 0.1 cm. We wanted to study the output signals when there is (a) progressive increase in the severity of defects and (b) single defect at locations along the direction of the beam. The word defect in this context can be synonymous to increase or decrease in the weight of an engineering structure [125]. Thus, removal of a pair of nut and bolt was considered as a single defect for simplicity to understand the basic principle as illustrated in the figure. For the experiments, 14 6-mm-diameter holes were first drilled in the beam at a spacing of 3 cm. Later, 14 pairs of nuts and bolts were fit into these holes as shown in Figure 6a. A radial-LSP sensor was attached to one end of the specimen and a piezoelectric diaphragm was attached in the middle, between the seventh and eighth nut and bolt pairs as shown in the figure. 

#### 3.3.2. Novel Principle

The basic principle involved was the supply of EM waves on the LSP sensor, which generated spoof SPP waves. These waves travel along the beam and a sudden presence of a circular shaped diaphragm sensor with a raised periphery, provided a means of confinement of these waves, which mostly travel ahead until they decay on the beam. For the experiments, the input was supplied at a distance of 0.7 mm above the LSP sensor whereas the output was captured at a distance of 0.7 mm above the catchment sensor as shown in the figure. Figure 6a shows 3D surface zones above the sensor/beam, which are considered to understand these two experiments. This phenomenon depends to a great extent on the conductivity of host structure and SPP travelling length. It is thus believed that the different material at the center will change the intensities of the waves at the location, which can be a point of interest for CMA engineering groups. However, electronics, physics, and medical researchers might find this principle quite unproductive as they are more interested in plasmonic properties and their variations. 

#### 3.3.3. Experimental Procedure

First, a baseline signal was measured to detect the default signal at no defect to the specimen; that is, the signal obtained when all the 14 nuts and bolts were fitted to the beam. Then, the first experiment was conducted by removing a pair of nut and bolt, one pair at a time. Each time, an output signal was captured until the final 14th pair of nut and bolt was removed. Next, the second experiment was conducted by removing single pair of nut and bolt at only one location along the direction of the beam while the rest of the holes are fitted with the other pairs of nuts and bolts. Thus, 14 output signals were captured one after another while 14 single defects were induced at locations from 1 to 14 by removing only one pair of nut and bolt when the other pairs are fitted.

#### 3.3.4. Discussions

The term ‘catchment’ is commonly used in civil engineering for a receiving zone, which stores water or fluids or gases coming from a single or multiple source away from it [126]. Thus, it is apt to use ‘catchment sensor’ to refer to piezo diaphragms in these experiments as this sensor captures or alters the incoming flow of surface/SPP waves. 

Figure 6b,c show RMSD curves for severity increase in defects and change in defect location, from the left to right end of the beam. Figure 6b shows an increasing trend of RMSD as defect magnitude increased from single defect until the severity of defects increased till the end of seven defects. However, the effect of increase in severity was not co-related beyond the catchment sensor; thus limiting the application scope of these two sensors. Figure 6c shows an increasing RMSD trend until the centre and then a decreasing trend. There was an approximate symmetry in the results when the defect is between the 1st and 7th pairs and, between the 8th and 14th pairs. The loss of weight at any location was reflected as a change in deformation at all locations including the centre, where the output signal was captured. 

These experimental results showed that the catchment method of acquisition of output signal at an adjacent sensor has a potential to become a necessary SHM tool in the future. More studies are required to understand the technique to better utilize such travelling surface waves for SHM.

## 4. Economical Miniaturized Arduino-Based Metamaterial Setup

### 4.1. Factors Effecting Economics in SHM

The first important aspect of economics in SHM is to reduce the cost of VNA, which are quite expensive of over US$ 40 K. However, it should be noted that in the initial years of research the cost of any equipment is usually higher than in the later years. There are several examples to prove that the cost gradually reduces over time. If we consider Hewlett Packard’s impedance analyzers [127] of the 1990s, they were very expensive compared to analyzers or LCR meters of the later decades [128]. The cost of VNA depends on (a) range of wavelength or frequency, and (b) measuring parameters. Thus, the cost hugely depends on the end user requirements, metamaterial designs, and models of analysers [129]. Further research is required to make it more affordable to developing countries such as India and China, where there is huge potential for metamaterials [130].

The next important aspect is the portability of devices, which plays a prominent role in SHM than in science or medicine. For example, a patient can walk in to get himself/herself admitted into the clinic for health issues but for SHM, the technology should walk-out from laboratories to the engineering structure under investigation. In the recent past, wireless devices are playing a major role by reducing all the complications involved in the laboratories [131]. 

The next important aspect is the simplification of the input source and the output probe to suit the end user requirement. Researchers have always explored metamaterials by changing input, elements and patterns to get optimum output signals to suit specific functions. Thus, it is quite common to use only limited features of expensive VNA for NDT/SHM. This paves a way to use an alternative and a limited device system such as Arduino kit, which is economical, portable, and lightweight compared to VNA. Thus, we adopted a highly economical and cost-effective Arduino Uno [132].

Another important aspect is the minuscule details of elements, which are generally important for physics or medical groups but are not that important for SHM. This is because CMA engineers prefer procurement of a commercially available cost-effective metamaterial sensor rather than designing and fabricating them. As seen earlier in Figure 3b, the longitudinal-LSP design is our newest entry, and it is highly economical (US$ 9) and has a larger sensing area than both the radial-LSP and spiral-LSP designs [77,102,113]. This was earlier validated as a force sensing resistor, but its resemblance to established LSP designs prompted us to adopt these sensors in our studies.

### 4.2. Arduino Meta Setup

**Figure 7:**
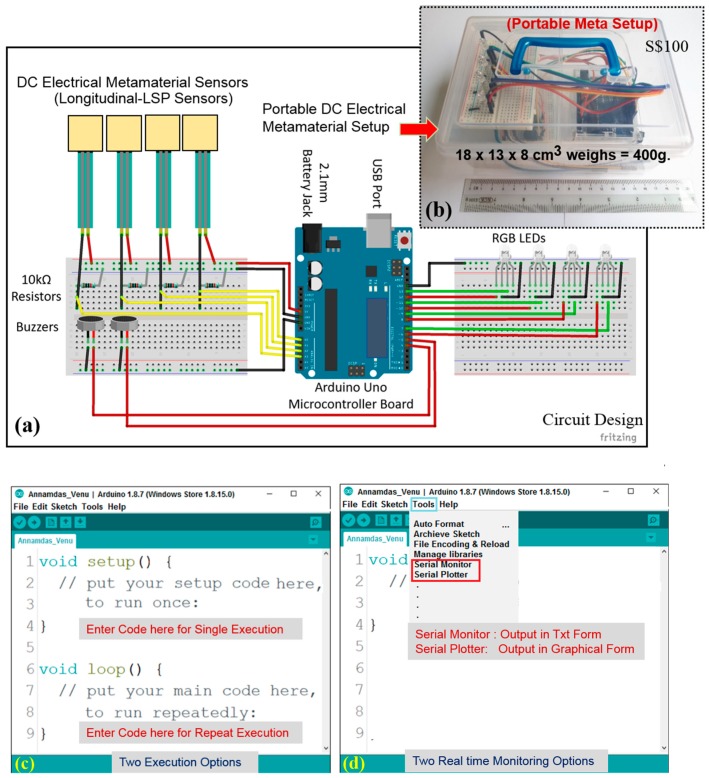
Portable Meta setup and software screen information. (**a**) Information of circuit diagram of kit; (**b**) photo of kit, in a ready to carry portable case; (**c**) code execution window screen; and (**d**) real time execution window screen.

Unfortunately, not just the analyzer but the far field based SSR and near field based LSP designs are expensive. Thus, a portable and economical setup which costs merely US$ 75(S$100) was developed for monitoring of load/pressure as shown in Figure 7. Figure 7a,b show a circuit diagram and a photo of a portable meta setup. It does not require expensive far field or near field setups as shown earlier in Figure 1b,c. This was encased in a meagre 18 × 13 × 8 cm casing with a weight of only 400 g. Moreover, if the circuit is printed on a PCB, the size and weight of this kit can be further reduced. Additionally, the longitudinal LSP design is also a highly flexible and water sealed sensor. Any amount of pressure applied on the top of it or on the structure to which it is bonded will result in a certain amount of resistance (Ω) in the sensor. In general, pressure increase will reduce the resistance, and when there is no applied pressure, the sensor will act like an infinite resistor (open circuit) without any current flowing. Thus, owing to this phenomenon the Arduino unit provided a DC electric voltage to the sensor instead of regular EM input. The output is a dimensionless value similar to scattered parameters which are also a dimensionless parameter. 

#### 4.2.1. Development

The kit consists of an Arduino Uno microcontroller board, a couple of breadboards (white color), four red (R)—green (G)—lack (B) LED, jumper wires, 10 kΩ exterior resistors, buzzers, battery holder and four longitudinal-LSP sensors. The kit is powered by a 9 V battery, using a battery holder, with a 2.1 mm plug which can be operated independently without any additional electrical connections. However, the input voltage supply is reduced to 5 V by a step-down transformer inside the board. Alternatively, the setup can be powered by a desktop or a laptop via a USB cable. The on-board microcontroller stores and runs a set of Arduino instructions in the form of programs, which are uploaded as per specific needs of the SHM [133].

For our experiments, we wrote several Arduino programs such as codes A, B, and C (see Supplementary file Tables S1–S3) using the freeware integrated development environment (IDE) software [134,135]. To activate the kit for the very first time, it has to be connected to the computer and the program code initiated in the application window as shown in Figure 7c. After which, the kit is ready to function on its own by blinking the on-board LED lights if it encounters a predefined load or pressure on the sensor. However, if the kit is connected to the computer, this facilitates data recording and display in the form of alphanumeric text or in the graphical form on the computer screen, based on the user’s selection of either ‘Serial Monitor’ or ‘Serial Plotter’ as shown in Figure 7d. Additionally, if the data is required but not necessarily displayed in real-time, then the PLX-DAQ software can be employed to record the obtained data from the sensor [136]. The software can capture the digital values as well as the actual time of recording into a Microsoft Excel worksheet. 

#### 4.2.2. Working Principle 

If such a sensor is subjected to a load below a prefixed threshold, a green LED light that is linked to that sensor will turn on, indicating the presence of the load on it. The threshold load is decided and set by the end user by changing the instructions in the code. Technically, when an LSP sensor is subjected to some pressure (input), the 10-bit ADC on board will convert the perceived analog voltage of a pin to a digital number (dimensionless output) [135]. The analogRead() function will be activated to read the perceived voltages of 0–5V and map them into integers between 0 to 1023. The 10-bit ADC feature allows the Arduino board to detect 1024 (210) discrete analog levels, i.e., load levels. This yields a resolution of 0.0049 V/unit or 4.9 mV/unit. However, when the load exceeds the threshold limit, the LED light will change from green to red and an alarm/buzzer will be activated. The program codes can be changed by the end user to suit the application or the threshold limits for activation of buzzers. For example, Arduino program code A operates for two longitudinal-LSP sensors and two buzzers connected to respective pins of the kit. These sensors when attached to a structure, behave as load monitoring tools to raise an alarm via related buzzer if the load exceeds certain predetermined threshold based on users’ choice of the external resistor, which has been pre-set at 10 ohms (Figure 7a).

#### 4.2.3. Optimization

**Figure 8:**
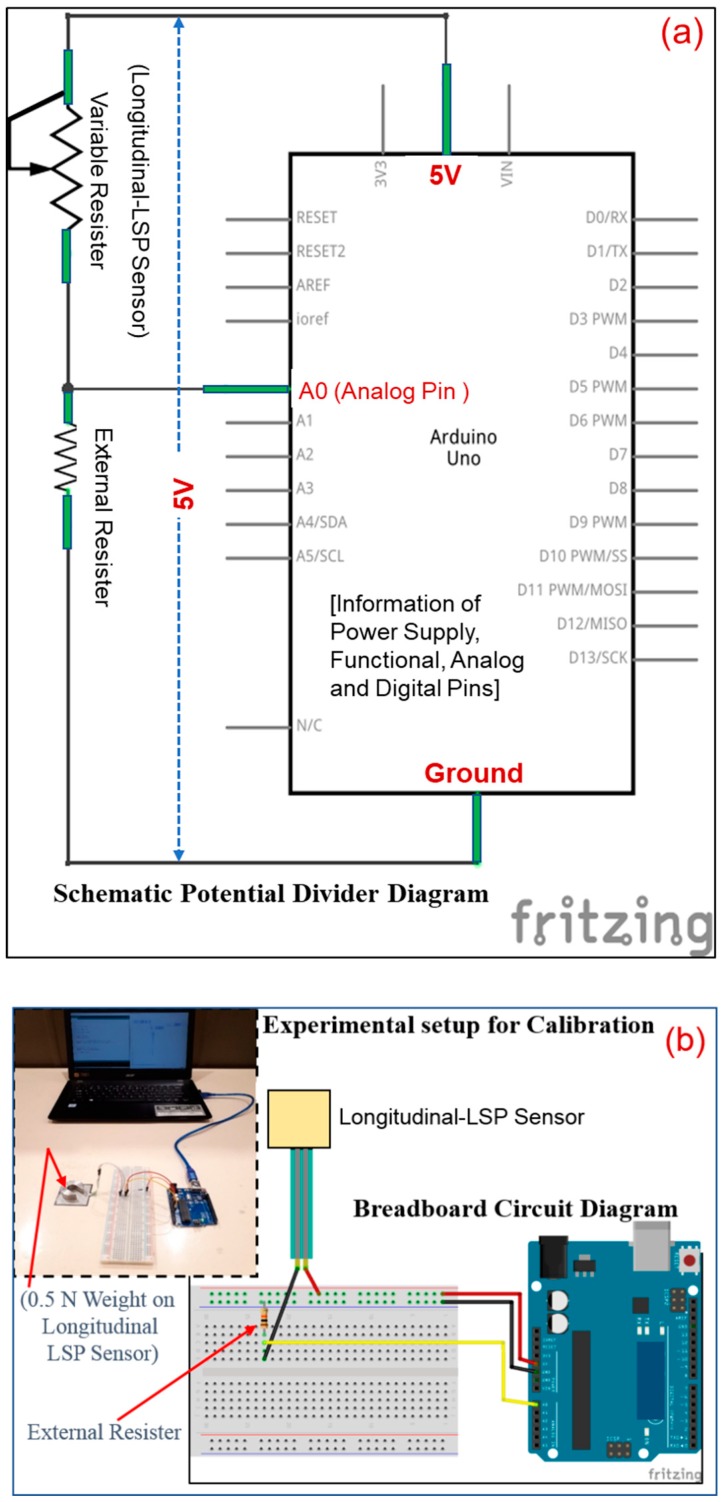

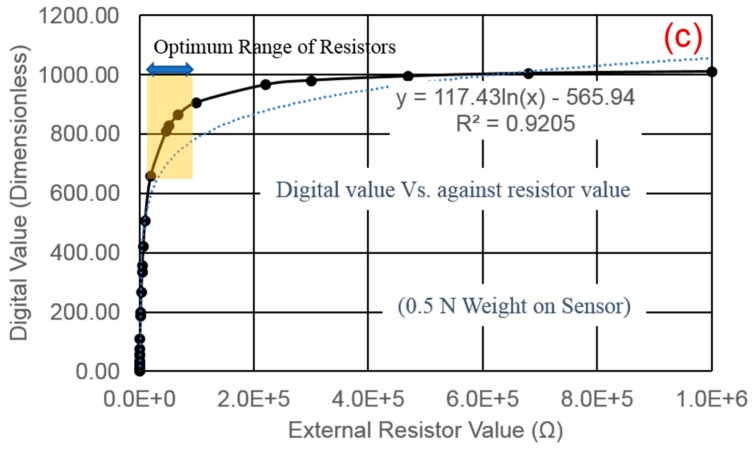
Optimization of external resistor for user desired force measurement system. (**a**) Schematic potential divider between external resistor and internal resistor; (**b**) experimental setup and circuit diagram; and (**c**) Digital values Vs external resistor.

The longitudinal-LSP sensor acts as a variable resistor whose resistance changes due to changes in load magnitude as stated earlier. Figure 8a shows the usage of input 5 V ( = $V_{in}$) in a series circuit, which gets divided between the variable resister $R_{LSP}$ and external resistor $R_{resistor}$. For a particular LSP sensor of the kit, the external resistor can be optimized, and the program can be modified for large number of sensors. The figure also shows internal details like analog pins, digital pins, etc. Figure 8b shows the experimental setup for the optimization, and the circuit diagram. The Arduino program code B for optimization was fed to the microcontroller via window screen, as shown in Figure 7b,c.

As stated earlier, in the absence of any load, the sensor behaves as an open circuit which will return meaningless value. Hence a nominal load of 0.5 N was first mounted on the sensor top and the code executed in real time for 5 seconds, during which a total of 50 digital values were recorded into Microsoft excel using the PLX-DAQ tool software. The average of these digital values was adopted as a mean digital output for the considered resistor at a nominal load. Then, the same test was repeated by varying the external resistors. A total of 30 resistors were tested which are 10 Ω, 22 Ω, 47 Ω, 100 Ω, 150 Ω, 200 Ω, 220 Ω, 270 Ω, 330 Ω, 470 Ω, 510 Ω, 680 Ω, 1 kΩ, 2 kΩ, 2.2 kΩ, 3.3 kΩ, 4.7 kΩ, 5.1 kΩ, 6.8 kΩ, 10 kΩ, 20 kΩ, 47 kΩ, 51 k, 68 kΩ, 100 kΩ, 220 kΩ, 300 kΩ, 470 kΩ, 680 kΩ, and 1 MΩ. The same code B was executed for each resistor $\left(\mathrm{say}\;R_{resistor}\right)$ and the mean digital output was obtained as shown in Figure 8c. All 30 data output points were joined by a logarithmic curve as shown in the figure. The expression and Regression value of the curve are printed on the figure. The formula to calculate the voltage across the LSP sensor is given by

(6) $$V_{LSP}=V_{in}\times \frac{R_{resistor}}{R_{resistor}+R_{LSP}}$$

Finally, the digital output of the LSP sensor under different external resistor was calculated as $V_{in}$ divided by 0.0049 V per unit. 

#### 4.2.4. Evaluation of Kit by Load Variations 

**Figure 9:**
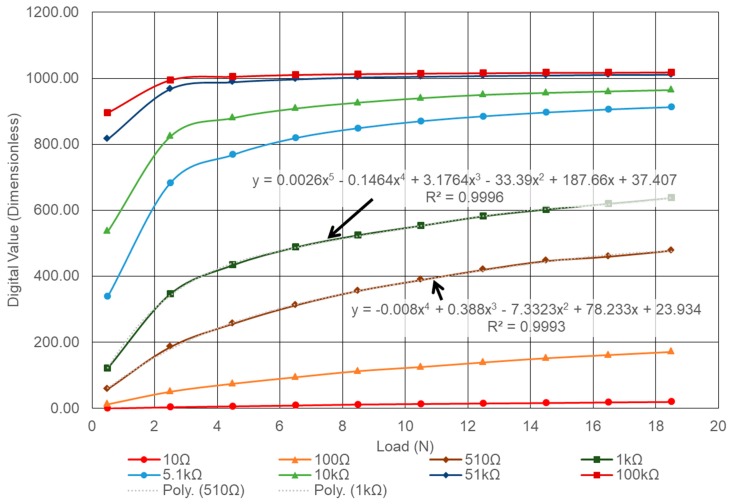
Force variation Vs. Load for various resistors.

Evaluation of the kit was carried out by conducting eight tests one after another, on eight different external resistors for various axial load increments by placing weights of 0.5 N, 2.5 N, 4.5 N, 6.5 N 8.5 N, 10.5 N, 12.5 N, 14.5 N, 16.5 N, and 18.5 N on top of the LSP sensor. Similar to the previous case, first a 0.5 N weight was gently placed on top of the sensor. The program code C was executed for the time window of 5 s during which 50 digital values were recorded into another Microsoft excel file as done previously. The mean digital output value was calculated for the load, and the same experiment was repeated by increasing the load. The entire process was repeated by changing the external resistors, and a graph of the obtained mean digital output values was plotted as shown in Figure 9. Table 1 shows the range of digital values from the smallest to the largest for all the resistors between the applied loads. There was no change in the output digital value when higher resistor was adopted for an increase in load. For example, the digital value does not have significant change when the load is above 2.5 N for the 100 kΩ resistor. Thus, such a resistor may not be suitable for determining the magnitude of load but will be suitable for mere detection of an applied load. On the other end of the spectrum, a similar trend can also be seen for the lower resistors, say 10 Ω, where there was a little change in the digital value for applied loads above 0.5 N. From the table, it is difficult to identify the magnitude of the applied load, as the digital output value remained more or less the same throughout. Furthermore, its digital curve (in color red) is at the bottom which is very close to the 0 value, i.e., parallel to the *X*-axis. This means that for lower resistors, the kit is not sensitive to either the presence or the magnitude of an applied load. However, the mean output digital value increased when the load increased especially for 510 Ω (brown color) and 1 kΩ (dark green color) as shown in the figure. Similarly, the difference of the maximum and minimum mean digital output value for the 510 Ω and 1 kΩ resistor is 418.88 and 515.15 respectively, which is considerably higher compared to the other resistors given in the table. The trend lines with associated mathematical expression and their regression values are also given in the figure. This wide range of output enables these resistors to be more sensitive, which allows detection of even a small load and any minor changes. Thus, the optimum resistors can be considered between 510 Ω and 1 kΩ. 

Such easy to build portable SHM setups are not only economical but offers a variety of programming options. Irrespective of whether such kits find any place in physics or medicine, they offer a variety of options for SHM of CMA engineering structures. 

## 5. Other Non-FO Smart Materials

There exist several non-FO smart materials such as electrorheological fluids, magneto-strictive materials, shape memory alloys, and—most importantly—piezoelectric type sensors [12,99,137,138]. It is difficult to present details of each material individually but it can be stated that the piezoelectric type sensors take a major share in the SHM market as they have superior actuation and sensing abilities compared to other sensors [131,139,140]. In the last 25 years, the usage of smart material sensors especially the application of piezo sensors increased in various techniques such as electromechanical impedance [123], ultrasonic guided waves [141,142], Lamb waves [143,144], vibration and damping control, energy harvesting, etc. [145,146]. The last decade has also seen various applications of smart materials in wired and wireless, contact and contactless sensing systems addressing several types of structural problems [118,123]. The most important applications of these sensors are found in crack detection, delamination of composites, fatigue, nut and bolt loosening, etc,. Especially for structures such as mechanical-blades, shafts, aeroplane wings, bridges, dams, slabs, beams, and columns, which are constantly subjected to various forms of internal and external loading resulting in strain variations. The applications of these smart materials in general and piezo sensors in particular, are too abundant to list for this review paper. Several review papers are available which [147,148,149,150,151,152] highlight the acceptability of piezoelectric type sensors, which are well established for engineering applications, unlike metamaterials. However, no single SHM technique or single type of sensor can be effective in addressing all SHM issues, especially if the structures are large and complicated. Thus, SHM using more than one technique with multiple smart materials is essential for monitoring life-sized infrastructures. Furthermore, the role of adjacent piezoelectric sensors for application of metamaterial sensing should be further investigated.

## 6. Summary

Several of the predominant sensors used for SHM are fiber-FBGs that are intermittently carved in FO cables, to transfer EM waves to and from the FBGs. These FBG sensors and FO cables need careful physical packaging but still, they occupy a lion’s share of the SHM market, especially for civil infrastructure. On the other hand, non-FO metamaterials such as LSP sensors are highly non-fragile as they are manufactured bi-material composites, with metal or plastic or polymer inscriptions on flexible and ultra-thin materials. They are bendable and twistable that range from linear ($0^{0}$) to double bend ($180^{0}$) shapes. Additionally, these LSP sensors do not require fragile packaging and can complement FO and FBG sensors in SHM. Additionally, no single smart material or SHM technique can ever address the complexities of CMA structures and fusion of such sensors along with existing sensing techniques is encouraged. 

The global demand for wired and wireless SHM is on the rise. Popular business strategists such as Technavio analysts, estimated the global SHM market to grow to US$ 3.38 billion in another 5 years, at a CAGR of 17.93%. Especially, the market of metamaterial is expected to grow rapidly at a rate of more than 22% and the market of piezoelectric materials is expected to accelerate at a rate of over 13%. At the same time, the global automation and robotics market in the automotive industry is expected to post a rate close to 8%. Hence, it is foreseeable that fusion of such smart materials with automation will increase the overall market enormously. 

In this context, this invited review presents several metamaterial designs along with our recent works, especially our new LSP design with portable meta setup and kit. Most of these designs are developed by the physics and electronics groups for sciences and medicine applications. Also, most of the present metamaterial designs have not been tried for CMA engineering structures. If any metamaterial or their breed have to be effective for SHM then their designs and implementation schemes need to be studied by CMA engineering researchers. The sensors need to be ultra-sensitive, portable, and water sealed for robust CMA engineering applications. Researchers are constantly improving their designs to make them suitable for rugged engineering monitoring. CMA and non-CMA researchers need to work closely to develop better designs for harsh environment engineering applications. Thus, this review paper draws attention to SRR designs of far field setup and LSP designs of near field setup. 

In the latter part of the paper, we introduced our longitudinal-LSP design to study sensing zone and water content effect. The study demonstrated the importance of exposing metamaterial sensors to the requirements of CMA engineering issues. Furthermore, a novel idea of utilizing the adjacent sensors such as piezoelectric diaphragms of a co-existing SHM technique, as catchment sensors are successfully presented. Finally, a miniaturized Arduino meta setup and kit, which is portable, economical and easy to launch compared to far field and near field is presented. This kit can operate with battery or via a portable computer. The data can be stored or displayed based on the choice of the end user. The user can adapt far field, near field or portable setups based on their requirements.

The efficiency of the meta setup and kit depends on the selection of the right operational resistor. High resistance values like 100 kΩ are not suitable to monitor the magnitude of the load but are suitable for detecting merely the presence of a load, due to their high sensitivity. On the other hand, low resistance values like 10 Ω are not suitable for either detection of the load magnitude or the presence of a load, due to their low sensitivity. However, the difference of the maximum and minimum mean digital output value for the 510 Ω and 1 kΩ resistor is 418.88 and 515.15 respectively, which is considerably high compared to other resistors. This wide range of output makes these resistors more sensitive, which allows detection of even a small load and any minor changes. Thus, the optimum resistors can be considered between 510 Ω and 1 kΩ.

## Figures and Tables

**Table 1:** Range of digital value for load range between 0.5 N to 18.5 N.

Resistor	10 Ω	100 Ω	510 Ω	1 kΩ	5.1 kΩ	10 kΩ	51 kΩ	100 kΩ
Range of digital value (difference between min to max applied load)	19.86	158.08	418.88	515.14	573.42	428.28	195.24	122.30

## Supplementary Materials

The following are available online at https://www.mdpi.com/1424-8220/19/7/1490/s1. Table S1: Program code for two metamaterial sensors with buzzers (Code A); Table S2: Program code for optimizing the value of external resister (Code B); Table S3: Program code for real time monitoring when connected to computer (Code C).

## Nomenclature

**Abbreviations:**	
ADC	Analog to digital converter
CMA	Civil, mechanical, and aerospace
CAGR	Compound annual growth rate
DC	Direct current
EM	Electromagnetic
FBG	Fiber Bragg grating
FO	Fiber optical
LCR	Inductance, capacitance, and resistance
LED	Light-emitting diode
LHM	Left hand metamaterials
LSP	Localized surface plasmon
MFC	Macro fiber composite
MRT	Mass rapid transit
NDT	Nondestructive testing
N-SRR	Nested split ring resonator
PCB	Printed circuit board
PDMS	Polydimethyl siloxane
PLX-DAQ	Parallax data acquisition tool
PZT	Lead zirconate titanate
RMSD	Root mean square deviation
R/G/B	Red/green/black
SHM	Structural health monitoring
SPP	Surface plasmon polariton
SRR	Split-ring resonator
TW	Thin wire
VNA	Vector network analyser
**Key Symbols:**	
Angle	ψ
Cartesian unit vectors	${\vec{u}}_{X}$, ${\vec{u}}_{Y}$, ${\vec{u}}_{Z}$
Distance vector	$\vec{r}$
Electric complex-valued amplitude vector	$\bar{E}$
Electric field	$\vec{E}$
External resistor	$R_{resistor}$
Gold	*Au*
Impedance	Z
Magnetic field	$\vec{H}$
Magnetic complex-valued amplitude vector	$\bar{H}$
Metal and dielectric variables	$P_{m}$, $P_{d}$
Permeability of the free space	$\mu_{o}$
Permittivity of the free space	$\varepsilon_{o}$
Relative permittivity of the dielectric material	ε
Resistor of LSP	$R_{LSP}$
Scattering	S
Scattering parameter (output/input: ports 2/1)	$s_{21}$
Voltages (internal: Arduino and external: LSP)	$V_{in},\;V_{LSP}$
Wave vector	$\vec{k}$
Wave propagation	K
Wavelength	λ
