# Supplementary material for: A Perspective of Non-Fiber-Optical Metamaterial and Piezoelectric Material Sensing in Automated Structural Health Monitoring
Source: Sensors (Basel). 2019 Mar 27;19(7):1490. doi: 10.3390/s19071490 (PMC6480363; doi:10.3390/s19071490)
Supplement: Supplementary file 1 [file sensors-19-01490-s001.pdf]

sensors MDPI

Supplementary file

Supplementary Table S1. Program code for two metamaterial sensors with buzzers (Code A)

```
/* (Program Name: Short Antenna Network Unit/ ShAnNU-A): Code A. Program to lit Green LED, but Red LED will start to blink above a predetermined threshold value, and also the buzzer will ring. */
const int META1 = A0; // Increase in compression
const int META2 = A1; // Increase in compression
const int ledG1 = 13; // For META1 Green LED
const int ledR1 = 12; // For META1 Red LED
const int ledG2 = 11; // For META2 Green LED
const int ledR2 = 10; // For META2 Red LED
const int buzz1 = 5; // Buzzer1
const int buzz2 = 4; // Buzzer2
const int ledState1 = LOW;
const int ledState2 = LOW;
const int buzzState1 = LOW;
const int buzzState2 = LOW;
int delay_time = 100; // Time interval to collect reading (in milliseconds)
int threshold_min = 1000; // Minimum threshold for green LED to light up
int threshold_press = 550; // Threshold for red LED
void setup() {
  Serial.begin(9600);
  pinMode(META1, INPUT);
  pinMode(META2, INPUT);
  pinMode(ledG1, OUTPUT);
  pinMode(ledR1, OUTPUT);
  pinMode(ledG2, OUTPUT);
  pinMode(ledR2, OUTPUT);
  pinMode(buzz1, OUTPUT);
  pinMode(buzz2, OUTPUT);
  Serial.println("CLEARDATA"); //clear excel sheet
  Serial.println("LABEL, Time, META1, META2 ");
void loop() {
  Serial.print("DATA,TIME, ");
  Serial.print(analogRead(META1));
  Serial.print(",");
  Serial.println(analogRead(META2));
 if (analogRead(META1) >= threshold_press) {
```

```
// Sensor 1
if (ledState1 == LOW) {
      ledState1 = HIGH;
    } else { ledState1 = LOW; }
    digitalWrite(ledR1, ledState1);
    digitalWrite(ledG1, LOW); }
  else if (analogRead(META1) >= threshold_min) {
digitalWrite(ledG1, HIGH);
    digitalWrite(ledR1, LOW);
  } else {
    digitalWrite(ledG1, LOW);
    digitalWrite(ledR1, LOW);   }
 if (analogRead(META1) >= threshold_press) {
 // Buzzer for Sensor 1
  if (buzzState1 == LOW) { buzzState1 = HIGH; }
    else { buzzState1 = LOW;}
    digitalWrite(buzz1, buzzState1); }
  else { digitalWrite(buzz1, LOW); }
/* Sensor 2. The, if – else statement here will make the LED light blink for above digital value ≥ threshold */
  if (analogRead(META2) >= threshold_press) {
    if (ledState2 == LOW) { ledState2 = HIGH;}
 else { ledState2 = LOW; }
    digitalWrite(ledR2, ledState2);
    digitalWrite(ledG2, LOW); }
  else if (analogRead(META2) >= threshold_min) {
    digitalWrite(ledG2, HIGH);
    digitalWrite(ledR2, LOW); }
  else { digitalWrite(ledG2, LOW);
    digitalWrite(ledR2, LOW); }
/* Buzzer for Sensor 2
  The if – else statement here will make the buzzer beep in pulse when the digital value is above the predetermined threshold.   */
  if (analogRead(META2) >= threshold_press) {
    if (buzzState2 == LOW) {
      buzzState2 = HIGH; } else { buzzState2 = LOW;
}
 digitalWrite(buzz2, buzzState2); }
  else { digitalWrite(buzz2, LOW); }
```

Supplementary Table S2. Program code for optimizing the value of external resister (Code B)

```
/* (Program Name: Short Antenna Network Unit/ ShAnNU-B): Code B for the study of the Metamaterial
Sensor under different external resistors. Readings will be taken for 5 seconds in Microsoft Excel. Take
average and plot graph */
const int META1 = A0;
int delay_time = 100; // in milliseconds
int next_interval = 10000; // time before program runs again (in milliseconds)
void setup() {
  Serial.begin(9600);
  Serial.println("CLEARDATA"); //clear excel sheet
  Serial.println("LABEL,Time,Sensor Value"); //label for excel
}
void loop() {
  for (int x = 1; x < 51; x = x + 1) {
    Serial.print("DATA,TIME, ");
    Serial.print(analogRead(META1));
    Serial.print(",");
    Serial.print("Reading ");
    Serial.println(x);
    delay(delay_time);
  }
  Serial.println("--- End ---");
  delay(next_interval); }
```

Supplementary Table S3. Program code for real time monitoring when connected to computer (Code C)

```
/*  (Program Name: Short Antenna Network Unit/ ShAnNU-C): Code C for real-time
monitoring of META */
const int FSR = A0;
int delay_time = 100;
void setup() {
  Serial.begin(9600);
  Serial.println("CLEARDATA"); // Clear Excel sheet
  Serial.println("LABEL,Time,Sensor Value"); // Label for Excel
}
void loop() {
    Serial.print("DATA,TIME, ");
    Serial.println(analogRead(FSR));
    delay(delay_time); }
```
